# Motivations, willingness, and gains: a qualitative study of experiences of helping behaviors among community-dwelling older adults in China using self-construal theory

**DOI:** 10.3389/fpubh.2026.1759118

**Published:** 2026-03-04

**Authors:** Wanhong Xiong, Chunyan Gu, Dan Wang, Xinyi Liu, Yu Luo

**Affiliations:** 1 School of Nursing, Army Medical University, Chongqing, China; 2 ShuangBei Community Health Service Center, Chongqing, China

**Keywords:** helping behavior, older adults, willingness, motivation, perceived gains

## Abstract

**Background:**

Prosocial behaviors are important public health strategies for decreasing older people’s social isolation and improving their social engagement and well-being. However, few studies have explored older Chinese people’s helping behaviors. Therefore, this qualitative study was designed to explore the experiences of helping behaviors among community-dwelling older adults using self-construal theory, focusing on their motivations, willingness, and gains.

**Methods:**

A descriptive qualitative study was conducted from July to September 2024 in Southwest China using semistructured, in-depth, in-person interviews. Participants were selected through purposive sampling method, and categories and subcategories were identified through content analysis method.

**Results:**

A total of twenty community-dwelling older adults participated in this study. Six categories and thirteen subcategories were extracted from the data analysis. The categories included (i) the individual self (intrinsic motivation and self-interested motivation); (ii) the relational self (driven by genetic factors and help people close to them); (iii) the collective self (responsibility and obligation and prioritizing collective interests); (iv) the beyond self (pure altruism and helping strangers discreetly); (v) perceived gains (intergenerational support, peer support, and positive emotions); and (vi) barriers to and facilitators of helping behaviors.

**Conclusion:**

The results emphasized that the intrinsic motivations for helping behaviors among older people were mainly benevolence and empathy. However, the motivations and willingness to engage in helping behaviors differed and were complex for different recipients. Perceived intergenerational support, peer support, and positive emotions could be protective factors for them in maintaining long-term helping behavior. Therefore, increasing helping behaviors in daily life should be considered an effective public health measure for older people to obtain family and social support and promote their mental health.

## 1 Background

By the end of 2022, the number of people aged 60 years and above in China had risen to 280 million, while those aged 65 years and above had reached 210 million, accounting for 19.8 and 14.9% of the total population, respectively (1). With the aging population, mental health issues among older peoplesuch as increasing social loneliness and declining social engagement-have significant effects on their quality of life and subjective well-being. Notably, the World Health Organization (WHO) defines active aging as the ability of older people to continue to participate in social, economic, cultural, spiritual and civic affairs, and to keep continuing positively to their families, peers, communities, and the country, which is one of the crucial measures in actively responding to population aging (2). Accordingly, the Chinese government has introduced a series of policies designed to integrate positive aging and healthy aging into the entire process of economic and social development, encouraging older people to actively participate in prosocial behavior to increase their quality of life and promote their well-being (3).

Prosocial behavior is defined as voluntary and intentional behaviors that benefit others, including family members, friends, or strangers (4), and it primarily encompasses helping behaviors, donating behaviors, volunteering behaviors, charitable giving behaviors, or sharing behaviors. A previous study indicates that as an innovative and effective measure for promoting public health and social harmony, helping behaviors can encourage older adults to engage in prosocial activities, thereby reducing social isolation, improving cognitive function, enhancing physical function, and decreasing the risk of dementia (5). Similarly, prosocial helping behaviors are closely associated with various outcomes in older people, including psychosocial benefits (e.g., decreased anxiety and depression, increased positive affect and psychological well-being, improved social support and social networks, etc.), physical outcomes (e.g., maintained functional independence and instrumental activities of daily living (IADL), increased the amount of activity and physical function, reduced mortality, etc.), and cognitive benefits (e.g., enhanced learning and memory ability) (5, 6). A national longitudinal study in China revealed that helping behaviors was negatively associated with older adults’depression, and the limitation of IADL significantly influenced the association between helping behaviors and life satisfaction (7). Compared with non-participants, those who engage in helping behaviors report higher satisfaction of competence satisfaction, greater fulfillment of basic psychological needs, more positive self-perceptions of aging, and better overall well-being (8, 9). Eisenberg (10) reported that positive affect was positively associated with individuals’ engagement in helping behaviors. Notably, helping behaviors impacted by social differentiation and economic interdependence play a core role in social cohesion through transcending close-knit networks and in-group boundaries, especially in multiethnic countries, and they can also promote social connection and reduce social isolation, thereby contributing to the well-being of both individuals and society (11). Moreover, previous studies reported that both formal and informal helping behaviors could reduce the risk of incidents of cardiovascular disease (12); the mortality salience was positively associated with helping behaviors during the COVID-19 pandemic, probably because a higher level of mortality salience prompted deeper reflection on death, which in turn led to a greater degree of helping behaviors (13). Additionally, both formal and informal helping were found to be beneficial for enhancing cognitive function among older adults, and the long-term postive impact of continuous helping behaviors on cognitive function among older adults had been confirmed, as goal-oriented helping behaviors could improve their problem-solving abilities and foster adaptive thinking (14). Neurobiological models (15) further revealed that the cognitive benefits of helping behaviors were mediated by the stimulation of downstream physiological pathways: the hypothalami–pituitary–adrenal (HPA) axis, the sympathetic nervous system (SNS), and the immune system.

Helping behaviors are influenced by factors such as sex, age, and family and social context (16). Evidence has shown that women often exhibit stronger social preferences and greater situational specificity in helping than men, which may be related to moral cultivation (17). Compared with younger adults, older people tended to show stronger in-group preferences, which indicated that they were more willing to donate money to domestic charities than foreign charities (18). Furthermore, helping behaviors also was significantly associated with the family context, such as parental psychological control, warmth, and helping values (19, 20). Compared with individuals with high levels of parental warmth, those with low levels were 2.2 times less likely to engage in helping behaviors (20). Similarly, as one of the crucial factors, the high quality of sibling relationships positively affects on individuals’ sharing behavior, helping behavior, or comforting behavior (21). Interestingly, previous study found that gossip has also been shown to effectively promot personal resource allocation and increase cooperative opportunities through reputation concerns, especially by increasing patterns of helping interaction at the group level (22).

Additionally, the cultural context plays a crucial role in helping behaviors. Individuals within individualistic cultures (predominant in Western countries such as the United States) tend to prioritize personal goals, whereas those within collectivist cultures (predominant in Eastern countries such as China) tend to prioritize group or collective goals (23). Accordingly, the motivation of Chinese volunteers, influenced by collectivism, focused on collectivist value expression, patriotism, national pride, and identity, while volunteers in the United States, influenced by liberalism, mainly exhibited the motivation of individualistic values (24). Previous studies found that couples in China’s collectivist cultural context repoted higher collectivism scores and greater relationship satisfaction than those in Malaysia’ individualistic cultural context (25); parental collectivism goals showed a significant positive association with Chinese adolescents’ helping behaviors (26). The traditional Confucian virtues, such as benevolence, empathy, and prosociality, have profoundly influenced the words and actions of the Chinese people for thousands of years (27), and they emphasize the interconnectedness of individuals within an in-group, and make each person’s well-being depend on the whole group (28). In conclusion, Chinese collectivism not only cultivates stronger sociocultural ties—which are strongly linked to greater helping behavior—but also promotes group cohesion and social harmony (29, 30).

Additionally, previous studies have revealed that self-construal is not only linked to socioculturs orientations (individualism versus collecivism) but also has a significant association with prosocial behavior (31). Self-construal theory revealed two types of self-construal: independent self-construal, prevalent in Western countries, which highlights the autonomous and independent individual, and interdependent self-construal, prevalent in Eastern countries, which emphasizes interpersonal relationships and social connectedness (32). Singelis (33) revelaed that the collectivist cultures exhibited a significant postive association with interdependent self-construal, whereas a negative association was observed with independent self-construal. Similarly, Zhang (34) found the interdependent self-construal among Chinses young people was linked to prosocial choices focused on benefiting others; Yaban (35) showed that within a collectivist cultural context, increasing independent self-construal could effectively promote the prosocial behavior among medical students in China. The tripartite self-construal theory by Brewer (36) further extends the self-construal theory by differentiating the interdependent self-construal into relational and collective self. At the individual level, the personal self is formed through interpersonal comparison and focuses on the self-interest; at interpersonal level, the relational self mainly arises through interpersonal feedback and emphasizes interpersonal connections with significant others; and at group level, the collective self is achieved through intergroup comparison and prioritizes the collective interests (36). Thus, this extended self-construal theory offers a more comprehensive explanation for multi-level differences in self-construa and better captures its dynamic changes across cultural contexts.

In conclusion, there are few studies on the experiences of helping behaviors among older Chinese people within a collectivist culture context. Therefore, this qualitative study was designed to explore the motivations, willingness, and gains of helping behaviors among community-dwelling older adults using the tripartite model of self-construal.

## 2 Methods

### 2.1 Study design

In this study, a descriptive qualitative research was conducted from July to September 2024 in Southwest China using semistructured, one-on-one, in-depth interviews, and the categories and subcategories were extracted through content analysis method. This study was approved by the Medical Ethics Committee of Army Medical University in Chongqing, China (Grant number: 20230901).

### 2.2 Participants

Participants were selected using purposive sampling method and the principle of maximum diversity. Prior to the study, researchers contacted the managers of the community health service center, explained the study’s purpose and procedures, and obtained their informed consent. Subsequently, recruitment posters detailing the study plans and participant criteria were placed on the center’s bulletin board to recruit sufficient participants. The inclusion criteria for participants were as follows: (i) community-dwelling older adults aged 60 years or older, (ii) residence in the main urban area of Chongqing, in China, for at least three months, (iii) normal cognitiv and communication ability, and (iv) signe written informed consent and participate voluntarily in this study. The exclusion criteria for participants were as follows: (i) had severe physical illnesses or were in an acute illness phase; (ii) had visual and/or hearing impairments; and (iii) withdrew from the study for personal reasons.

### 2.3 Data collection

Data were collected through semistructured, in-depth interviews from July to September 2024 in Southwest China. Following a comprehensive literature review and in-depth team discussion, the interview guidelines were finalized (shown in Table 1). Prior to each interview, the researcher sufficiently explained the study details to participants, and informed consents were obtained from particitants after confirmed their comprehensive understanding. If telephone interviews, the participants provided oral consent. Moreover, all participants were informed of their right to withdraw at any time for any reason. Interviews took place in a private room, either at the community service center or the participants’ home, and were conducted by a trained female nursing doctor candidate experienced in qualitative research. With the participants’ permission, the interview process and the expressions and nonverbal behavior of the interviewees were recorded by a voice recorder and the researcher. Importantly, to ensure confidentiality, each interviewee was anonymous and coded as *P*1, *P*2, ……*P*n. In addition, the principle of data saturation with no new topic or issues emerging was used to determine the sample size (37). In this study, data saturation was reached after twenty interviews, and three additional participants were interviewed to confirm this. Consequently, data from twenty participants were included in the final analysis.

**Table 1:** Semistructured interview guideline.

Interview questions
1. Did you help others? Please describe the details.
2. Why did you help them?
3. What did you perceive some gains through helping others? What’s your feelings?
4. Who are you willing to help? What are you willing to do to help them? Why?
5. What factors do you think might promote your helping behaviors?
6. What factors do you think might prevent you from helping others?

### 2.4 Data analysis

The interview materials were transcribed verbatim into Microsoft Word 2003 documents within 24 h after each interview, and NVivo 11.0 software was used to assist in data management and analysis (38). We adopted content analysis (39) to analyze the data. First, two researchers independently and immersively read the original materials to gain comprehensive understanding of the participants’ meanings. Second, during this process, meaning units were identified, condensed and encoded, with coded remaining as close as possible to the original text. Third, codes were clustered. Interrelated codes were then grouped into subcategories which were subsequently identified and synthesized to categories. Any differences in this process were resolved through discussion or by consulting a third researcher. Furthermore, any hypotheses were avoided throughout analysis process, and all data were secretly stored.

### 2.5 Rigor

The validity and trustworthiness of this study were measured by Guba and Lincoln’s criteria of credibility, confirmability, transferability, and dependability (40). To ensure credibility, in the interview process, the interviewers were conducted by researchers with adequate training and extensive experience in qualitative methods, and open-ended interview guidelines, and both verbal and nonverbal data were recorded to obtain more information about the research questions from the participants. To ensure confirmability, the study adhered to established procedures for the descriptive qualitative research and content analysis, including detailed audio recording, timely data transcription, repeated immersion in the original materials, identifying and coding of meaning units, and the development of categories and subcategories. Transferability was supported by timely data transcription, the meaning units being encoded remaining as close as possible to the original text, and detailed result description with using representative citations. To ensure dependability, two researchers independently and repeatedly read and continuously compared the data, and identified and coded the meaning units using the original text as much as possible. Furthermore, trustworthiness was enhanced by following the qualitative content analysis processes of decontextualization (condensing and coding) and recontextualization (creating categories and categories on various levels) (39).

## 3 Results

### 3.1 Sociodemographic characteristics

In total, twenty community-dwelling older adults were enrolled in this study, including nineteen face-to-face interviews and one telephone interview. The average interview time was 32.00 ± 10.09 min (ranging from 22.35 to 67.17 min). The demographic characteristics of participants were presented in Table 2.

**Table 2:** Sociodemographic characteristics of participants (*n* = 20).

Characteristics		*n*	%
Age (Mean±SD, min to max, years)		69.1 ± 5.88(60.00 to 80.00)	
Gender	Female	10	50%
Education level	Primary school degree	3	15.00%
	Middle school degree	8	40.00%
	High school degree	3	15.00%
	Associate degree	5	25.00%
	Bachelor’s degree	1	5.00%
Marital status	Married	12	60.00%
	Divorced	1	5.00%
	Widowed	4	20.00%
	Remarriage	3	15.00%
Living condition	Living alone	4	20.00%
	Living with spouse	10	50.00%
	Living with offspring	2	10.00%
	Living with both spouse and offspring	4	20.00%
Previous occupations	Worker	8	40.00%
	Farmer	3	15.00%
	Police	1	5.00%
	Teacher	2	10.00%
	Driver	2	10.00%
	Self-employment venture	2	10.00%
	Senior engineer	1	5.00%
	Manager	1	5.00%
Monthly income (CNY)	1,000–2,999	4	20.00%
	3,000–4,999	11	55.00%
	≥5,000	5	25.00%
Current working condition	Retirement	18	90.00%
	Re-employment after retirement	2	10.00%
Self-assessment of health status	Good	3	15.00%
	Better	10	50.00%
	General	1	5.00%
	Poor	6	30.00%
Number of chronic diseases	0	4	20.00%
	1	10	50.00%
	2	3	15.00%
	>3	3	15.00%
Interview style	Face-to-face	19	95.00%
	Telephone interview	1	5.00%
Interview Time (Mean ± SD, min to max, minutes)		32.00 ± 10.09 (22.35 to 67.17)	

**Figure 1:**
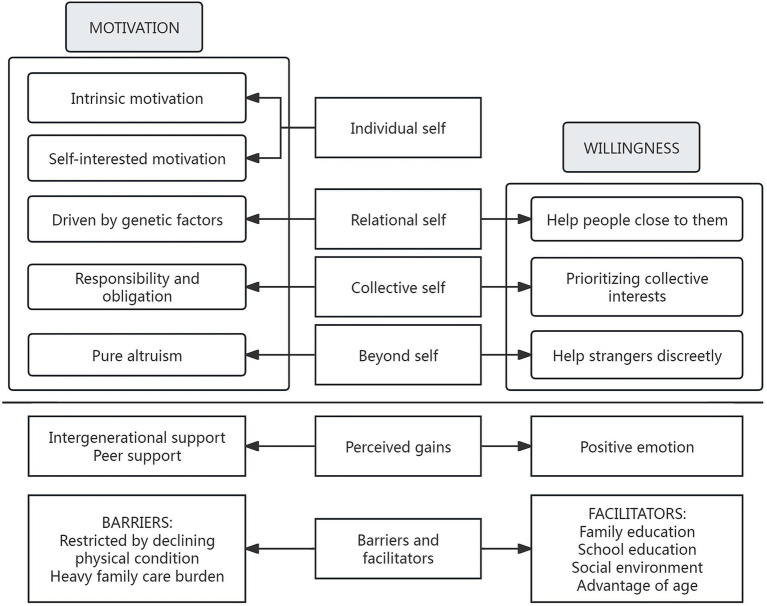
The motivations, willingness and gains of helping behaviors among community- dwelling older adults.

The following six categories were extracted from data: (i) the individual self (intrinsic motivation and self-interested motivation), (ii) the relational self (driven by genetic factors and help people close to them), (iii) the collective self (responsibility and obligation and prioritizing collective interests), (iv) the beyond self (pure altruism and help strangers discreetly), (v) perceived gains (intergenerational support, peer support, and positive emotions), and (vi) barriers to and facilitators of helping behaviors, as shown in Figure 1. The coding details of representative quotes, subcategories, and categories were shown in Supplementary Table 1.

### 3.2 Category 1: The individual self

#### 3.2.1 Intrinsic motivation: benevolence and empathy

Intrinsic motivation refers to an individuals’ inherent tendency to engage in activities that they hold intrinsic interest for (41). In this study, the intrinsic motivation, such as personality traits, and inherent or emotional satisfaction, focused on older people’s an innate propensity to participate in helping behaviors. Most participants explained that their helping behaviors were motivated by an inner sense of benevolence and empathy. When older adults encountered people suffering from serious disease (e.g., cancer) or browsed online news about the devastating impact of war on family and societies, they often display empathy and sympathy, even sometimes to the point of tears, those emotional response, in turn, stimulated them to engage in more helping behaviors, such as comforting or donation. Some participants reported that teaching people older than themselves to use social media (e.g., mobile phones) to promote social engagement and reduce loneliness. They also viewed this behavior as a way to perform good deeds.

*“I do not consider profit or loss when I am assisting people. I believe I ought to act with fundamental benevolence, and doing so will also help my descendants accumulate good deeds.*” *(P10).*

*“Their economic situation is more difficult than mine, the job of their children is not good, and the family income is very low. They are worthy of sympathy.” (P16).*

#### 3.2.2 Self-interested motivation: promoting self-improvement

Notably, participation in helping behaviors could help older adults cultivate positive attitudes, learn to experience other’s lives, enrich their thoughts, grow more independent, and promote self-improvement including problem-solving ability and communication skills, which in turn supports them in achieving life goals and career success.

*“When my family members are in fear and in need of help, I will unhesitatingly give them a hand and help them find solutions to problems…Even if they are under financial pressure, I will still help them and give them hope. In the process of helping them, it also helps me improve the capacity for dealing with complex problems and my communication skills…I feel that my life is wonderful and fulfilling.” (P5).*

### 3.3 Category 2: The relational self

#### 3.3.1 Driven by genetic factors

“Break the bones and tendons” and “blood is thicker than water” both imply that blood relatives should be closer to one another than nonrelated individuals are. Given the strength of kinship, older adults stated that they considered it their duty to assist their siblings and other relatives.

*“My brother received a cancer diagnosis. In addition to providing him with money every month for treatment, I look after him at the hospital.” (P4).*

*“I will definitely help my relatives, definitely…The responsibility I should take, I will do it without hesitation. The help I should offer, I will do it without hesitation…Family affection is-blood is thicker than water…Relatives should always help each other, and that is an instinct.” (P8).*

Some participants expressed that caring for their grandchildren, including providing daily life care, playing games with them, picking them up from school, and engaging in other activities, was a major part of their lives and made up for their guilt caused by the fact that they were too busy to look after their own little children when they were young. Moreover, grandparents are crucial in fostering moral values for their grandchildren, including encouraging them to share, express gratitude, respect older people, and show filial the piety toward their parents. They firmly believe that the maxim “to be a good person, to cultivate virtue first” was ingrained in their minds, and that upholding strong moral principles was crucial for improving interpersonal relationships and collaborate with others, which was essential for both professional and personal success. In addition, nearly all of the participants stated that helping care for grandchildren and lessening the load on their own children were customary practices among Chinese people. They had strong willingness to take care of their grandchildren because their own grandparents had helped them when they were young.

*“I tell my son that being a good man should come before anything else. I actively teach him what is right or wrong in daily life, which helps shape his sense of right and wrong. In my opinion, benevolence is crucial.*” *(P8).*

*“When I was little, my grandparents raised me because my parents were working. So now I also help take care of my grandson…this is passed down from generation to generation, helping to take care of the grandchildren.” (P6).*

#### 3.3.2 Help people close to them

Nearly all participants reported that their helping behaviors were primarily directed toward people they knew, such as their neighbors or others close to them. These behaviors mainly included buying groceries for older people living alone, offering dietary advice to sick friends to help them maintain a healthy diet, assisting them in solving problems, and so on. However, participants indicated that how much help they gave largely depended on how close they were to the person. Moreover, when supporting their close friends, they also considered the other’s willingness to accept help, sometimes even worrying about “Harm the self-esteem,” leading to misunderstandings and strained relationships.

*“How close we are will dictate how much I can support him…If the relationship is close, I will help him without hesitation…However, if it’s not that close, I can choose to do it or not.” (P2).*

### 3.4 Category 3: The collective self

#### 3.4.1 Prioritizing collective interests

The participants emphasized the collective as the core and prioritized national and/or collective interests, indicating that they would set aside their personal interests to uphold national and/or collective interests when the two conflicted. As a member of the group, it was their responsibility and obligation to contribute to the collective, and they reported that preserving group interests was tantamount to preserving their own interests. Furthermore, a sense of collective honor was another important reason for participants safeguarding their collective interests, because individual and collective interests rise and fall together.

*“When individual interests conflict with collective interests, we will certainly prioritize collective interests. As a nursing manager, I will consider the collective interests of the nurses and stand up for their interests with reason.” (P10).*

*“I will give priority to the interests of the country and the collective… As a police officer… I am also willing to sacrifice my life for the country and the collective when necessary.” (P14).*

#### 3.4.2 Responsibility and obligation

Additionally, for some participants, especially those in professions such as police, sustaining social harmony is not just an act of kindness but also a product of professional habits within their professional fields. It was their responsibility and professional practice to encourage virtue and to help resolve social problems.

*“As a factory worker, it was my responsibility to put out the fires and reduce the factory’s losses.” (P7).*

*“As a police officer, maintaining social harmony is my professional duty, which motivates me to help others in need and resolve social disputes even now that I am retired.” (P14).*

### 3.5 Category 4: The beyond self

In this study, beyond self refers to the transcendence of self-centered considerations in an individual’s cognition, emotions, and behaviors, and it was introduced to explain the helping behaviors displayed by older people toward strangers.

#### 3.5.1 Pure altruism: being voluntary and unpaid

Pure altruism refers to a specific preference characterized by exclusively outcome-oriented motivation and entirely devoid of any personal satisfaction or direct utility derived from the giving act itself (42). The participants’ pure altruism was demonstrated primarily by the fact that they actively and voluntarily helped others without expecting anything in return. They felt that “it was not worth mentioning,” implying that offering help is as natural as eating and sleeping, and that there is no need to discuss it with others because such acts are entirely selfless, voluntary, and unpaid. Influenced by traditional Chinese culture and moral values, people believe that helping behaviors should be done quietly and that it is unnecessary to let others know who is providing assistance.

*“I always offer assistance to those in need. However, in my opinion, these actions do not need to be known by others.” (P8).*

*“Whether it is a country or a family, it is truly good when everyone is well…Everyone should be well… If your life is good while others are struggling, or if others’ lives are good while yours is difficult, society will not be harmonious…Everyone should be in harmony.” (P9).*

#### 3.5.2 Help strangers discreetly

Regretfully, most participants were hesitant about assisting strangers because they lacked trust in them. Herd mentality meant their helping behaviors were influenced by family members or friends, and they preferred to provide assistance through official channels, such as community volunteer programs or organized charitable donations. Additionally, they tended to help those in need, such as people with disabilities, pregnant women, or young children, rather than healthy beggars. Furthermore, older people exhibited a prosocial preference for the domestic population, prioritizing the welfare of their own country and fellow residents.

*“Before helping strangers, I will assess whether he is being dishonest based on his story of hardship, his clothing, and his appearance. I do not help strangers readily.” (P2).*

*“Currently, some beggars hide their feet to pretend to be disabled and beg…This is deceptive, so I cannot possibly help them.” (P7).*

### 3.6 Category 5: Perceived gains

#### 3.6.1 Intergenerational support

Notably, although older adults stated that their helping behavior toward family was voluntary and unpaid, they still received additional support in return such as family and intergenerational support, particularly emotional support, which further increased their happiness and well-being.

*“My grandson often cares about me. Although he does not give me any money which I also do not need anymore, he always calls to check on me… Right now, psychological comfort and satisfaction are all my needs…When I was ill, he called and said ‘Grandpa, are you okay? Are you going to see a doctor?’…” (P13).*

#### 3.6.2 Peer support

Peer support is a valuable strategy for assisting others by sharing supporters’ experiences, offering disease knowledge, and providing emotional support (43). Among older people with cancer, supportive peer relationships were formed in specific environment, and older adults reported that peer support could provide them with positive emotions, optimistic attitudes toward life, and coping skills, which helped reduce anxiety symptoms, decrease symptoms burden, and increase confidence and self-efficacy in coping with diseases.

“*As a small family, we share, comfort, and support one another. There was a patient with terminal cancer who remained very optimistic and often shared that outlook with us…In that way, we support each other.”(P15).*

#### 3.6.3 Positive emotions

“The fragrance remains in the hand that gives the rose.” Giving love and support to others greatly affects both the giver and receiver, leading to feelings of fulfillment, personal growth, and happiness. Some older adults emphasized that if they did not extend a hand to people in need, they would feel a sense of guilt.

*“Helping others is happiness. I often tell my son that if you help someone…such as sharing your favorite food with others, you will fell happy.” (P10).*

*“If I did not help people in need, I would feel guilty. So, I always donate money to the people who are ill.” (P20).*

### 3.7 Category 6: Barriers and facilitators

#### 3.7.1 Barriers to helping behaviors

##### 3.7.1.1 Restricted by declining physical condition

The participants’ physical health had a major effect on their helping behaviors. Older people experience physical declines such as low energy and joint discomfort in their limbs that limit their mobility, and these bodily changes make them feel unable to engage in helping behaviors. For example, they would choose to call an emergency number rather than help a fallen older people stand up. Additionally, their daily activities became largely restricted to moving between their home and the hospital; they shifted focus away from their families and communities to prioritize self-care and receiving regular medical treatment.

*“I was diagnosed with prostate hyperplasia five years ago, and I needed a permanent urinary catheter, which made it difficult to leave the house to help others.” (P12).*

##### 3.7.1.2 Heavy family care burden

In contemporary Chinese society, family structure has changed dramatically, Compared with the traditional extended-family model in which multiple generations lived together, most older people now live onlu with their spouses, providing mutual support and care, especially when one becomes ill or faces other serious health challenges. Unfortunately, these heavy familial responsibilities restricted their activities outside the home, reduced opportunities for social interactions, and impeded their engagement in helping behaviors.

*“My wife and I live together, and she was recently diagnosed with Parkinson’s disease, with her mobility and balance declining. I could not go out because I had to take care of her entirely by myself.” (P13).*

#### 3.7.2 Facilitators of helping behaviors

##### 3.7.2.1 Family education

According to the participants, they were persistently instilled with kindness and helpfulness by their parents, and their helping behaviors were strongly influenced by parental actions, such as comforting others, donating money or labor, and sometimes making personal sacrifices to help others. Additionally, their helping behaviors were significantly enhanced by schooling and social education, such as learning the spirit of Lei Feng and participating in patriotic activities like visiting martyrs’ graves.

*“My father is a doctor, and he often teaches us to help others. And my mother is also very kind. She often prioritizes others’ interests over her own and puts others people first. Whether it’s relatives, friends, or neighbors, she always helps them…Wherever I can, I try my best to help others.” (P9).*

*“My father’s helping behavior has imperceptibly influenced my own…Helping others helps yourself…Family education is very important.” (P10).*

##### 3.7.2.2 School education

Throughout the interviews, older people mentioned that education on revolutionary heroes and moral exemplars in schools (e.g., the spirit of Lei Feng, a person likes to help others) systematically constructed their cognitive framework of altruistic and dedication. Furthermore, through the infiltration of Confucian benevolent in courses such as “filial piety and fraternal duty” and “humaneness and love,” a continuous internal motivation for helping others has been sustained. Being kind to others is deeply rooted in their minds and as a traditional element of the Chinese culture, it is seen as something to be upheld and passed down.

*“The education we received in schools, such as traditional Confucianism and Taoism, deeply influenced our helping behaviors…The Confucian idea of ‘being kind to others’ is a tradition belief of the Chinese nation, emphasizing kindness and tolerance in how people should treat one another.” (P14).*

*“Doing good deeds brings me great happiness… That’s exactly what Lei Feng did… We should all learn from Lei Feng.” (P3).*

##### 3.7.2.3 Social environment

With the rapid development of the information age, older adults can often browse videos about helping behaviors on TV, in newspapers, and even through apps like Tik Tok on their smartphones. The widespread circulation of such videos and information also provides a favorable social environment for fostering a positive ethos of “when one is in difficulty, all sides come to help.”

*“In newspapers, on TV, radio, or mobile phones, videos and information about helping behaviors are shared… When seeing reports of helping others, I think that the helper is truly admirable… For example, if someone helps others through his own effort… that is certainly very admirable.” (P8).*

##### 3.7.2.4 Advantage of age

Despite declining physical health, most participants stated that their helping behaviors were not hindered by age. For them, being older was also an advantage, as it allowed them to accumulate more life experience, strengthen their problem-solving skills, improve their communication abilities, and enhance their social competence. Importantly, they experienced less stress in daily life and work than before, and they have enough time and financial means to engage in helping activities.

*“Aging will not stop me from helping others. Compared with young people, I have plenty of life experience and stronger interpersonal communication skills.” (P15).*

## 4 Discussion

This qualitative study explored in depth the motivations, willingness and perceived gains of helping behaviors among community-dwelling older adults using tripartite self-construal theory, and six categories were extracted: the individual self, the relational self, the collective self, the beyond self, perceived gains, and barriers to and facilitators of helping behaviors, which was inconsistent with the findings of Ramezani (44). The results highlighted that the intrinsic motivations of older adults’helping behaviors were benevolence and empathy, while the self-interest motivation promotes their self- improvement. However, older people exhibited distinct motivations and willingness depending on the recipients: a strong in-group preference for their family members and friends, driven by genetic factors; prioritizing collective interests for collectives or nations, driven by a sense of responsibility and obligation; and voluntarily but discreetly offering help for strangers, driven by purely altruistic motivation. Notably, although it was repeatedly emphasized that helping behaviors were selfless, older adutls still perceived some gains, including intergenerational support, peer support and positive emotions.

Community-dwelling older adults reported that the motivations of their helping behaviors were core moral qualities of benevolence and empathy. The significant role of moral values and beliefs has been confirmed, showing that individuals with a strong moral identity more are more inclined to engage in helping, donating, volunteering, charitable giving, or sharing (45). In traditional Confucian culture, benevolence is regarded as a core virtue that profound influences individuals’ morality, ethics, and behavior (46). Influenced by this traditional cultures, older adults emphasized that they were persistently instilled with values of benevolence and helpfulness by their parents since childhood. Notably, emotional empathy was identified as a strong predictor of helping behaviors; compared with younger adults, older adults exhibited higher levels of emotional empathy and greater engagement in of helping behaviors (47). Previous studies have shown that downward social comparisons can stimulate empathy, which in turn predicts increased helping behaviors (48). In this study, older adults stated that encountering individuals with disabilities or poor economic conditions evoked their compassion and emotional empathy to offer help. Additionally, this study found that the direct self-interested motivation of older people’s helping behaviors was to enhance their own abilities, rather than to seek economic returns. This results are consistent with the results of Gaber (49), which reported that participating in volunteer activities helps older people improve their communication skills, provide more opportunities for social interaction and learning, encourage self-reflection on health behaviors, and foster positive experiences. These findings indicated that through engaging in helping behaviors, older adults can not only gain emotional satisfaction and psychological comfort but also improve their social adaptability. Future research should further explore how helping behaviors influence older adults’ social networks, relationship quality, and overall longevity.

In this study, the findings revealed that community-dwelling older adults exhibited different motivations and willingness of helping behaviors depending on recipients. Driven by genetic bonds and kinship, they expressed a stronger willingness to help family members, especially their grandchildren. In addition to formal helping (e.g., donating, volunteering), it also included informal helping behaviors, such as helping a fallen older people, assisting individuals with disabilities, and engaging in housework or grandparenting (50). A systematic review by Danielsbacka (51) revealed that grandparenting could not only reduce family burdens and maintain family harmony, but also enable older people to obtain enough intergenerational support and improve their health outcomes, including improving sleep quality, reducing loneliness, and increasing social support and well-being (52, 53). Notably, influenced by reduced social networks, older people tend to withdraw from social relationships and devote themselves to family network at the core, which mainly includes housework, grandparenting tasks, and spouses support. The socioemotional selectivity theory explains that with advancing age, older adults narrow their social scope to prioritize social interaction quality, and prefer interactions with spouses, siblings, relatives, and close friends, who also become their primary targets of their helping behaviors (54). Moreover, they exhibited a stronger kin-selection orientation in their monetary and time donations, which may reflect an expectation of the reciprocity, as they also relied on and hoped to receive family support, and they also showed greater in-group favoritism, which predicted a tendency to help and cooperate with in-group members (55). Additionally, community-dwelling older people also expressed that they prioritized collective interests, viewing them as a personal responsibility and obligation. Jiao (56) reported that individuals consistently influenced by collectivist culture and values displayed more altruistic behavior and greater tolerance for unfair behavior than those influenced by individualistic culture. Yu (57) revealed that social responsibility, as a universal social attribute, stimulated people to transcend self-interest, prioritized collective or group interests, and increase their willingness to engage in helping behaviors. Therefore, policymakers should actively build communities guided by collective values and promote collectivist cultural education to strengthen older people’s sense of collective and social responsibility, thereby improving their motivation to engage in helping behaviors.

In addition, in this study, older adults also engaged in altruistic behavior-voluntarily helping others without expectation of reward, but even so, during these helping behaviors, they also perceived positive emotions and peer support. Previous studies (58, 59) have shown that prosocial behavior has a significant positive effectiveness on participants’ psychological benefits and social connectedness, especially in terms of positive affect, a postive association being observed among them. Huang (60) reported that social support is a strong positive predictor of prosocial behaviors with positive emotions mediating this relationship. These findings could be explained by that higher perceived social support foster a more positive cognition and understanding of the external environment and a more positive response to others’ needs, thereby promoting greater engagement in helping behaviors (61). However, older adults often acted with discretion toward strangers, showing caution in donating money or other helping behaviors, which probably be explained by lack of social trust and fear of being deceived. Ramezani (44) also confirmed that a lack of social trust was an important factor for older people engaging in informal spontaneous helping behaviors. Decreasing physical function and mental capacity result in a reduced ability to process complex information, to distinguish truth from falsehood, and to engage in self-protection, which make older adutls easy target for fraud and deception, especially online deception. Therefore, providing enough social support and enhancing their capacity to cope with deceptive behaviors were important for improving their social trust and promoting their motivation and willingness to engage in helping behaviors.

Notably, the results revealed that helping behaviors among community-dwelling older adults were limited mainly by their declining physical condition and heavy family care burden. Specifically, advancing age is associated with a progressive decline in physical function, including reduced muscle strength, diminished hearing and vision function, wear of bones and joints, impaired mobility and balance ability, and an increased prevalence of chronic diseases (62). Previous studies found that perceived physical vulnerability was positively associated with willingness to donate (63); regular physical exercise can enhance not only older people’s physical function (e.g., mobility, balance, and muscle strength) but also their social skills, interpersonal communication ability, and willingness to engage in social activities and helping behaviors (64). In addition, caregiver burden is significantly associated with adverse physical, psychological, emotional, social and financial outcomes, as well as reduced quality of life (65). Compared with male caregivers, older female caregivers are more likely to experience such burden, especially physical burden (66). Therefore, with the population aging, policymakers shoud pay more attention to the physical and mental health of older caregivers and establish an intelligent social support system to provide enough psychological and emotional support for them.

There study has several limitations. First, the sample was drawn from a single urban area in Chongqing, which may restrict the generalizability and transferability of the findings to other populations (e.g., rural, institutionalized, or hospitalized older adults). Second, although a risk of selection bias could been carried from purposive sampling, it could be controlled by the principle of maximum diversity across participants’ demographic characteristics, to some extent. Finally, as the quality of the semistructured interviews dependents mainly on the researcher, the results may have be impacted by several potential confounding factors.

## 5 Conclusion

Based on the tripartite self-construal theory, this qualitative study was designed to explore in depth the motivations, willingness and perceived gains of helping behaviors among community-dwelling older adults, and the findings provided new insights into how and why older adults engage in helping behaviors in their daily lives. The results emphasized that the intrinsic motivations of helping behaviors were mainly originated from benevolence and empathy. However, older people exhibited distinct motivations and willingness depending on the recipient: (i) For family and friends, a typical in-group preference was observed, driven by genetic and relational bonds; (ii) For collectives or the nation, helping behaviors were motivated by a sense of responsibility and obligation, and (iii) For strangers, helping was voluntarily and discreetly offered, driven by purely altruistic cognition. Notably, although the selflessness of helping behaviors was repeatedly emphasized, older adutls still perceived intergenerational support, peer support and positive emotions through these behaviors. Therefore, policymakers should formulate some policies and provide financial support to encourage older adutls to actively participate in helping behaviors, which was play crucial roles in increasing their quality of life and overall well-being.

## Data Availability

The raw data supporting the conclusions of this article will be made available by the authors, without undue reservation.
