# Supplementary material for: Motivations, willingness, and gains: a qualitative study of experiences of helping behaviors among community-dwelling older adults in China using self-construal theory
Source: Front Public Health. 2026 Mar 4;14:1759118. doi: 10.3389/fpubh.2026.1759118 (PMC12995783; doi:10.3389/fpubh.2026.1759118)
Supplement: Supplementary file 1 [file Table_1.docx]

**Supplementary Table 1 The coding details of representative quotes, sub-categories, and categories**

| **Categories** | **Sub-categories** | **Representative quotes** |
| --- | --- | --- |
| 1. The individual self | Intrinsic motivation: Benevolence and empathy | *"I do not consider profit or loss when I am assisting people. I believe I ought to act with fundamental benevolence, and doing so will also help my descendants accumulate good deeds." (P10)*  *“Their economic situation is more difficult than mine, the job of their children is not good, and the family income is very low. They are worthy of sympathy.” (P16)* |
|  | Self-interested motivation: Promoting self-improvement | *“When my family members are in fear and in need of help, I will unhesitatingly give them a hand and help them find solutions to problems…Even if they are under financial pressure, I will still help them and give them hope. In the process of helping them, it also helps me improve the capacity for dealing with complex problems and my communication skills…I feel that my life is wonderful and fulfilling.” (P5)* |
| 2. The relational self | Driven by genetic factors | *"My brother received a cancer diagnosis. In addition to providing him with money every month for treatment, I look after him at the hospital.” (P4)*  *“I will definitely help my relatives, definitely…The responsibility I should take, I will do it without hesitation. The help I should offer, I will do it without hesitation…Family affection is-blood is thicker than water…Relatives should always help each other, and that is an instinct.” (P8)*  *"I tell my son that being a good man should come before anything else. I actively teach him what is right or wrong in daily life, which helps shape his sense of right and wrong. In my opinion, benevolence is crucial.” (P8)*  *"When I was little, my grandparents raised me because my parents were working. So now I also help take care of my grandson…this is passed down from generation to generation, helping to take care of the grandchildren.” (P6)* |
|  | Help people close to them | *“How close we are will dictate how much I can support him…If the relationship is close, I will help him without hesitation…However, if it’s not that close, I can choose to do it or not.” (P2)* |
| 3. The collective self | Prioritizing collective interests | *“When individual interests conflict with collective interests, we will certainly prioritize collective interests. As a nursing manager, I will consider the collective interests of the nurses and stand up for their interests with reason.” (P10)*  *“I will give priority to the interests of the country and the collective... As a police officer… I am also willing to sacrifice my life for the country and the collective when necessary.” (P14)* |
|  | Responsibility and obligation | *"As a factory worker, it was my responsibility to put out the fires and reduce the factory's losses.” (P7)*  *"As a police officer, maintaining social harmony is my professional duty, which motivates me to help others in need and resolve social disputes even now that I am retired.” (P14)* |
| 4. The beyond self | Pure altruism: Being voluntary and unpaid | *“I always offer assistance to those in need. However, in my opinion, these actions don't need to be known by others.” (P8)*  *“Whether it is a country or a family, it is truly good when everyone is well…Everyone should be well… If your life is good while others are struggling, or if others' lives are good while yours is difficult, society will not be harmonious…Everyone should be in harmony.” (P9)* |
|  | Help strangers discreetly | *“Before helping strangers, I will assess whether he is being dishonest based on his story of hardship, his clothing, and his appearance. I don’t help strangers readily.” (P2)*  *“Currently, some beggars hide their feet to pretend to be disabled and beg…This is deceptive, so I can't possibly help them.” (P7)* |
| 5. Perceived gains | Intergenerational support | *"My grandson often cares about me. Although he doesn't give me any money which I also don't need anymore, he always calls to check on me… Right now, psychological comfort and satisfaction are all my needs…When I was ill, he called and said ‘Grandpa, are you okay? Are you going to see a doctor?’…" (P13)* |
|  | Peer support | *"As a small family, we share, comfort, and support one another. There was a patient with terminal cancer who remained very optimistic and often shared that outlook with us…In that way, we support each other.”(P15)* |
|  | Positive emotions | *“Helping others is happiness. I often tell my son that if you help someone…such as sharing your favorite food with others, you will fell happy.” (P10)*  *"If I didn’t help people in need, I would feel guilty. So, I always donate money to the people who are ill.” (P20)* |
| 6. Barriers and facilitators | | |
| 6.1 Barriers to helping behaviors | Restricted by declining physical condition | *"I was diagnosed with prostate hyperplasia five years ago, and I needed a permanent urinary catheter, which made it difficult to leave the house to help others.” (P12)* |
|  | Heavy family care burden | *“My wife and I live together, and she was recently diagnosed with Parkinson's disease, with her mobility and balance declining. I could not go out because I had to take care of her entirely by myself.” (P13)* |
| 6.2 Facilitators of helping behaviors | Family education | *“My father is a doctor, and he often teaches us to help others. And my mother is also very kind. She often prioritizes others' interests over her own and puts others people first...Whether it's relatives, friends, or neighbors, she always helps them…Wherever I can, I try my best to help others.” (P9)*  *“My father’s helping behavior has imperceptibly influenced my own…Helping others helps yourself…Family education is very important.” (P10)* |
|  | School education | *“The education we received in schools, such as traditional Confucianism and Taoism, deeply influenced our helping behaviors…The Confucian idea of ‘being kind to others’ is a tradition belief of the Chinese nation, emphasizing kindness and tolerance in how people should treat one another.” (P14)*  *"Doing good deeds brings me great happiness... That's exactly what Lei Feng did... We should all learn from Lei Feng.” (P3)* |
|  | *Social environment* | *“In newspapers, on TV, radio, or mobile phones, videos and information about helping behaviors are shared... When seeing reports of helping others, I think that the helper is truly admirable... For example, if someone helps others through his own effort,, that is certainly very admirable.” (P8)* |
|  | Advantage of age | *"Aging will not stop me from helping others. Compared with young people, I have plenty of life experience and stronger interpersonal communication skills.” (P15)* |
